# The effect of heterospecific and conspecific competition on inter-individual differences in tungara frog tadpole (*Engystomops pustulosus*) behavior

**DOI:** 10.1093/beheco/arac109

**Published:** 2023-01-07

**Authors:** Cammy Beyts, Maddalena Cella, Nick Colegrave, Roger Downie, Julien G A Martin, Patrick Walsh

**Affiliations:** The Roslin Institute and R(D)SVS, Easter Bush Campus, University of Edinburgh, Easter Bush, Midlothian, EH25 9RG, UK; Digital Futures, Warnford Court, 29 Throngmorton Street, London, EC2N 2AT, UK; Institute of Ecology and Evolution, School of Biological Sciences, University of Edinburgh, West Mains Road, EH9 3JT, UK; Institute of Biodiversity Animal Health and Comparative Medicine, R205A Level 2, The University of Glasgow, G12 8QQ, UK; Department of Biology, Marie-Curie Private, University of Ottawa, Ontario, K1N 9A7, Canada; Institute of Ecology and Evolution, School of Biological Sciences, University of Edinburgh, West Mains Road, EH9 3JT, UK

**Keywords:** animal personality, behavioral syndrome, competition, conspecific, heterospecific, inter-individual differences, tadpole, variance partitioning

## Abstract

Repeated social interactions with conspecifics and/or heterospecifics during early development may drive the differentiation of behavior among individuals. Competition is a major form of social interaction and its impacts can depend on whether interactions occur between conspecifics or heterospecifics and the directionality of a response could be specific to the ecological context that they are measured in. To test this, we reared tungara frog tadpoles (*Engystomops pustulosus*) either in isolation, with a conspecific tadpole or with an aggressive heterospecific tadpole, the whistling frog tadpole (*Leptodactylus fuscus*). In each treatment, we measured the body size and distance focal *E. pustulosus* tadpoles swam in familiar, novel and predator risk contexts six times during development. We used univariate and multivariate hierarchical mixed effect models to investigate the effect of treatment on mean behavior, variance among and within individuals, behavioral repeatability and covariance among individuals in their behavior between contexts. There was a strong effect of competition on behavior, with different population and individual level responses across social treatments. Within a familiar context, the variance in the distance swam within individuals decreased under conspecific competition but heterospecific competition caused more variance in the average distance swam among individuals. Behavioral responses were also context specific as conspecific competition caused an increase in the distance swam within individuals in novel and predator risk contexts. The results highlight that the impact of competition on among and within individual variance in behavior is dependent on both competitor species identity and context.

## Teaser

Competing individuals need to reduce the negative impacts of competition. When competing against a superior competitive species, tungara frog tadpoles differ in their behavior, allowing them to specialize on a narrower subset of resources. When competing with their own species, tadpoles all behave very similarly, promoting coordinated resource use. Tadpoles therefore vary the strategy they use to offset the negative impacts of competition depending on who they are competing with.

## INTRODUCTION

Among-individual (co)variation in the behavior of animals is now well characterized (Sih et al. 2004; Réale et al. 2010; Dingemanse and Dochtermann 2013). Animal personality is used to describe instances of among individual variation in the mean behavioral response of a population (e.g. variation in the average daily distance each individual travels). Whereas behavioral syndromes describe among individual correlations of a behavioral response measured across discrete ecological contexts (e.g. the daily average distance individuals travel in a familiar vs a novel context). Animal personality is thought to be driven by intrinsic differences in state between individuals and may be maintained by genetic variation, phenotypic changes to the genotype to different environments, and equal fitness payoffs associated with different behavioral strategies (Stamps 2007; Wolf et al. 2008; Mathot et al. 2012).

In ecology, the niche specialization hypothesis uses comparable statistical and biological concepts to understand how conspecific and heterospecific competition for food and space may drive among individual differences in dietary preference, to allow limited resources to be partitioned among individuals (Bolnick et al. 2003; Araújo et al. 2011). These behavioral and ecological frameworks are now becoming integrated through the social (Bergmüller and Taborsky 2010; Montiglio et al. 2013) and behavioral niche (Kent and Sherry 2020) hypotheses, which predict that conspecific and heterospecific competition will increase among individual differentiation in behavior to reduce conflict over resources. These multi-species interactions are important for understanding the proximate causes of animal personality and behavioral syndromes as well as individual interactions which promote the co-existence of conspecifics at high density and co-occurrence of multiple species with similar resource needs (Bolnick et al. 2003; Pfennig et al. 2006, 2007; Briffa andSneddon 2016; Kent and Sherry 2020; Sherry et al. 2020).

**Figure 1:**
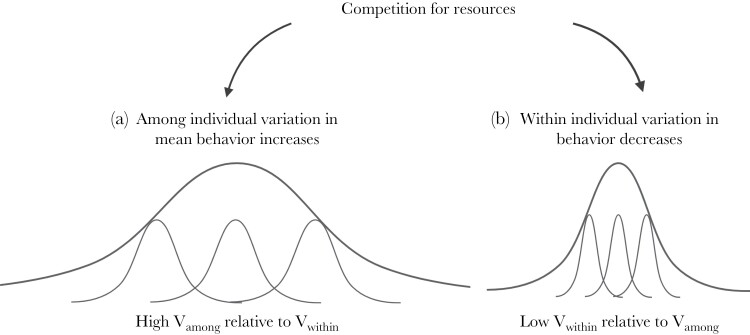
Conceptual illustration of two potential effects on individual level differences in behavior in response to competition. The thin lighter lines are the behavioral responses of individuals and the darker thicker lines are the behavioral response of the population. The repeatability (*R* = Vamong/Vamong + Vwithin) of plots (a) and (b) is the same. In plot (a) individuals diverge in their behavioral strategy via an increase in among individual variation in mean behavior. In plot (b) individuals diverge in their behavioural strategy via a decrease in within individual variation. Created with BioRender.

Under competition, there may be two ways in which individual changes in behavior may lead to consistent differences among individuals in their behavior and resource use. (Figure 1). Individuals may diverge in their average behavior, so that a broader range of behavioral strategies can be used to acquire a more diverse set of resources (Figure 1a; Preisser et al. 2009; Prati et al. 2021). For example, less competitive individuals may be forced to forage at less optimal times of day or in less profitable foraging locations (Rychlik 2005; Frere et al. 2008; Harrington et al. 2009; Wauters et al. 2019). This would be detectable as an increase in the variance among individuals as individuals diverge in their average behavior.

Alternatively, competition for resources may also affect how consistent individuals are in their behavioral strategy, by influencing how variable individuals are within themselves (Stamps et al. 2012; Westneat et al. 2015). Here individuals may specialize in a particular microhabitat by showing greater consistency in their foraging behavior (Figure 1b; Beaulieu and Sockman 2012; Newell et al. 2014; Sherry et al. 2020). For example, each individual may specialize in foraging at a specific time of day within the most optimal foraging hours for that species. Therefore, individuals would diverge in their behavior as they become less variable within themselves (Dingemanse and Dochtermann 2013) and would be detectable as a decrease in the variance within individuals. Consequently, under competition individuals may behave differently from each other either because variation in behavior among individuals increases or because variation within individuals decreases. The repeatability statistic can be used to understand when competition may be driving differences among individuals and where individuals also show high consistency in their behavior within themselves (Bell et al. 2009). The individual variance components used to calculate repeatability can then be used to determine whether it is variability among or within individuals which is responsible for this change (Bell et al. 2009; Nakagawa and Schielzeth 2010; Jäger et al. 2019). Repeatability will be high when variance among individuals is high relative to within individual variance or when within individual variance is low relative to among individual variance (Nakagawa and Schielzeth 2010; Dochtermann and Royauté 2019).

Behavioral repeatability may also change across ecological contexts (Stamps and Groothuis 2010; Arvidsson et al. 2017; Mitchell and Houslay 2021). This is because novel and risky contexts may result in potentially bolder or more cautious behaviors compared to familiar, low risk contexts (Carter et al. 2013; Perals et al. 2017; Kelleher et al. 2018). An individual’s perception of risk may further be dependent on the extent and type of competition they are exposed to during development (Urszán et al. 2015; Han and Dingemanse 2017; He et al. 2017; Castellano et al. 2022). Increased competition for resources may mean that individuals which are in greater need of resources may be prepared to take more risks and travel further distances in unfamiliar contexts (McNamara and Houston 1987, 1994; Anholt and Werner 1995, 1998). Therefore, patterns of behavioral repeatability may be influenced by the competitive environment as well as be context specific.

Different competitive environments may also favor specific combinations of behavioral responses across different ecological contexts (Bell 2005; Dingemanse et al. 2007; Fischer et al. 2016). In the absence of competition, there may only be a weak association between an individual’s behavioral response in familiar, novel or risky contexts (Bell and Sih 2007; Dingemanse et al. 2007). However, exposure to competition may require individuals to up- or down-regulate their foraging activity across a range of contexts to secure additional resources (Bergmüller and Taborsky 2010). Consequently, conspecific and heterospecific competition may cause individuals to change their behavior across multiple contexts which would be detectable as a behavioral syndrome, that is, correlations between context specific behavioral responses at the among individual level (Garamszegi and Herczeg 2012; Dingemanse and Dochtermann 2013).

In this study, we investigate the effect of conspecific and heterospecific competition in the tungara frog tadpole (*Engystomops pustulosus*) which can be frequently found inhabiting the same temporary pools with the whistling frog tadpole (*Leptodactylus fuscus*) in Trinidad (Downie and Nicholls 2004). Both species have a similar development time of three weeks and occupy a similar ecological niche, suggesting a high level of resource overlap (Murphy et al. 2018; Santana et al. 2019; Atencia et al. 2020). The superior competitive ability of *L. fuscus* is thought to be attributed to its larger starting size and higher activity rates (Downie and Nokhbatolfoghahai 2006; Downie et al. 2008). Amphibian larvae represent an ideal life stage and group of organisms in which to investigate the effects of competition on animal personality and behavioral syndromes (Urszán et al. 2015, 2018; Castellano et al. 2022). Across a variety of species, tadpoles compete with both conspecifics and heterospecifics for access to resources to fuel fast growth and development prior to metamorphosis and many of these interactions involve asymmetrical competition between species (Werner 1992; Bardsley and Beebee 2001; Richter-Boix et al. 2004, 2007; Smith et al. 2004; Ramamonjisoa and Natuhara 2017; Castellano et al. 2022).

The behavioral traits we investigated were the total distance individuals traveled in a set period across three ecological contexts, namely, the total distance traveled in a familiar context, novel context and predatory risk context. We will refer to the total distance traveled in each context as activity behavior, exploration behavior and predatory risk-taking behavior respectively. In a familiar context, we predicted that conspecific and heterospecific competition would increase the repeatability of swimming behaviors through an increase in among individual variance in mean behavior and/or decrease in within individual variance. We also predicted that the repeatability of behavior would differ between the three contexts and that behavioral differentiation would be greater under heterospecific compared to conspecific competition. Finally, we predicted that competition with conspecifics and heterospecifics would lead to correlations in behavioral responses between contexts at the among individual level, which may not be present in the absence of competition.

## METHODS

### Study species and collection sites

**Figure 2:**
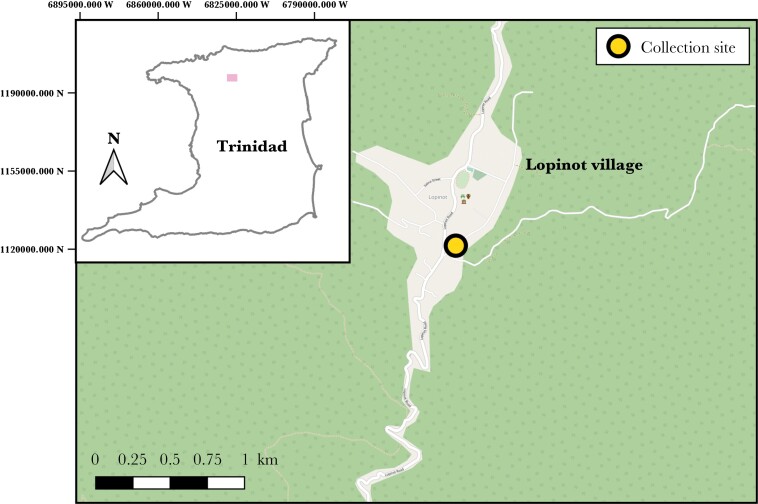
Map displaying the collection site of *Engystomops pustulosus* and *Leptodactylus fuscus* foam nests in Lopinot Village, Trinidad. Map created using the Free and Open Source QGIS.

We collected a total of 31 *E. pustulosus* and 25 *L. fuscus* foam nests from Lopinot Village, Trinidad (DMS: 10°41ʹ21.7″N, 61°19ʹ26.9″W) between June and July 2019, across four separate collection trips (see section 2.2). We collected nests from pools located along a 400-m length of road where both species are known to co-occur (Downie 2004; Figure 2). We placed each *E. pustulosus* nest into a separate container (dimensions: 145 × 100 × 55 mm) containing water from the collection site and each *L. fuscus* nest into containers lined with a damp paper towel. As *L. fuscus* tadpoles rely on heavy rainfall to be washed into larval pool, they can suspend their development after hatching in the absence of water (Downie 1984, 1994). However once submerged in water, their development continues as normal. Consequently, eight of the 25 *L. fuscus* nests collected had already hatched but had not developed beyond Gosner stage 27–28 (Gosner 1960).

We transported the nests back to the William Beebe Tropical Research Station, “Simla”, (DMS10°41ʹ30.7″N 61°17ʹ26.4″W) located in Trinidad’s Northern Range, within 2 h of collection. *E. pustulosus* tadpoles emerged from their eggs between 24 and 48 h after collection. *L. fuscus* tadpoles from nests which had not already hatched were more variable in their emergence time, emerging between 24 and 96 h post collection. We exposed nests and tadpoles to a 12.5L: 11.5D photoperiod and ambient temperatures ranging between 23.4 °C and 27.9 °C (24.8 °C ± 0.02 SD).

### Experimental design

**Figure 3:**
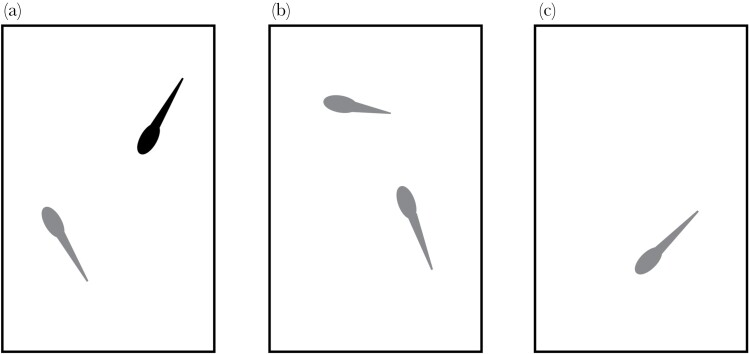
Illustration of treatment regimes. (A) Heterospecific treatment containing one *E. pustulosus* (grey) and one *L. fuscus* (black) tadpole. (B) Conspecific treatment containing one focal (full tail) and non-focal (shortened tail) *E. pustulosus* tadpoles. (C) No-competition treatment containing a solitary *E. pustulosus* tadpole.

We established three ontogenetic treatment groups to examine the impact of conspecific and heterospecific competition on *E. pustulosus* tadpole behavior: 1) The “heterospecific treatment” contained one *E. pustulosus* tadpole and one *L. fuscus* tadpole (Figure 3A); 2) the “conspecific treatment” contained two *E. pustulosus* tadpoles, each from different nests (Figure 3B) and 3) the “no competition treatment” which contained an *E. pustulosus* tadpole housed in isolation (Figure 3C).

We repeated the experiment over four consecutive batches, corresponding to the four collection trips. In each batch, we collected between 3 and 10 *E. pustulosus* foam nests 3–4 days before assigning tadpoles to their experimental treatments. *L. fuscus* nests were collected slightly earlier, 5–6 days prior to treatment assignment, due to the longer development time of *L. fuscus* eggs.

Within each batch, we assigned 15 focal *E. pustulosus* tadpoles to each of the three treatment groups, which were chosen at random from two *E. pustulosus* foam nests which hatched on the same day. This was to ensure that focal *E. pustulosus* tadpoles were the same age across each of the three treatment groups. Within the conspecific treatment, tadpoles from the two *E. pustulosus* nests were assigned as the focal or non-focal tadpole. To avoid potential weaker competitive dynamics among related individuals (Pakkasmaa and Aikio 2003; Yu and Lambert 2017), we obtained the non-focal tadpole in the conspecific treatment from the other nest to ensure that competitors were not siblings. To distinguish focal tadpoles in the conspecific treatment, we removed 1/3 of the non-focal tadpole’s tail under MS-222 anesthesia (Segev et al. 2015; Clarke et al. 2019). This distinguishing feature quickly disappeared, due to tail regeneration, so we subsequently distinguished focal tadpoles by visual differences in snout-vent-lengths that became apparent 3–4 days after the treatment commenced.

Focal and non-focal *E. pustulosus* tadpoles were at Gosner stage 25–26 when they were added to their treatment groups. The *L. fucus* tadpoles were more developed (Gosner stage 27–28) than the *E. pustulosus* tadpoles in the heterospecific treatment, reflecting the natural circumstances of *L. fuscus* tadpoles in the wild typically entering breeding pools at a later stage of development (Downie 1984; Downie and Nicholls 2004).

**Figure 4:**
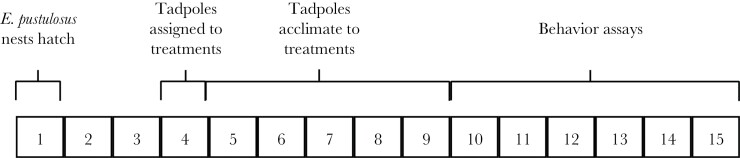
Summary timeline for an experimental batch. Numbers represent 24-h days.

We housed tadpoles in all treatments in plastic tanks (dimensions: 100 × 65 × 37 mm), filled with 150 ml of de-chlorinated, aerated tap water. We covered the tank sides in opaque tape, so tadpoles were not influenced by visual cues from tadpoles in adjacent tanks. We fed each tadpole in batches two through four with 7 mg of ground fish food (TetraMin Tropical Fish Food Flakes) per day in the first week and 10 mg in the second week. Due to a smaller initial size, we fed tadpoles in batch one with 3 mg of food in the first week and 7 mg in the second week. As there were two tadpoles per tank in the conspecific and heterospecific treatments, the amount of food provided in these treatments was doubled. We left tadpoles undisturbed for 5 days following their assignment to treatments to allow them to acclimate and develop under their new social environment before starting behavioral assays. The experiment took 15 days from *E. pustulosus* hatching to the completion of the behavioral assays (Figure 4). This represents 60% of the larval period under ideal growth conditions. Tadpoles were returned to their sites of origin within 7 days of completing their final behavioral assay.

Across the four experimental batches, we collected data from 54 tadpoles in the no-competition treatment, 56 focal tadpoles in the conspecific treatment and 51 tadpoles in the heterospecific treatment. 40 tadpoles across the three competition treatments died during the experiment which we did not include in the final tadpole count (Supplementary material). We returned unused tadpoles and nests to their sites of origin within 7 days of collection.

### Behavioral assays

We recorded the behavior of each tadpole in three behavioral assays named the activity, exploration, and predator risk-taking assays to record the total distance tadpoles swam in familiar, novel and predator risk contexts respectively. We recorded each individual’s behavior on six separate occasions over six consecutive days for each of the three assays. We recorded assays in the same order (activity, exploration, and predatory risk-taking) to limit the carry-over effects of the more disruptive exploration and predatory risk-taking assays (Bell 2013). There was a total of 960, 993 and 909 trials recorded from the no-competition, conspecific and heterospecific treatments respectively. We removed partial recordings from tadpoles that died before completing all 6 trials and recording errors (e.g. due to power outages) which were identified in 36/2898 trials.

We recorded all assays using one of four Canon Legria HF R86 camcorders, which we fixed in position (height: 450 mm) above the activity tanks and exploration/predatory risk-taking arenas. We could film two tadpoles in separate, adjacent tanks/arenas simultaneously under one camera. The tanks/arenas could be positioned and removed from under the camera but were held in a fixed position during trials to assist with automated tracking software (see section 2.7). We filmed all the assays in a room adjacent to the laboratory where we performed husbandry procedures, under the same temperature and lighting conditions, to ensure that tadpoles would be undisturbed during filming.

### Activity assay

To measure activity levels in a familiar context, we filmed the movement of focal *E. pustulosus* tadpoles in their home/rearing tanks over a 10-min period. In the heterospecific and competition treatments, we removed non-focal tadpoles and placed them in a small cup of water from their home tank prior to starting the assay. All tadpoles were left undisturbed for 10 min prior to filming to allow them to acclimatize.

### Exploration assay

**Figure 5:**
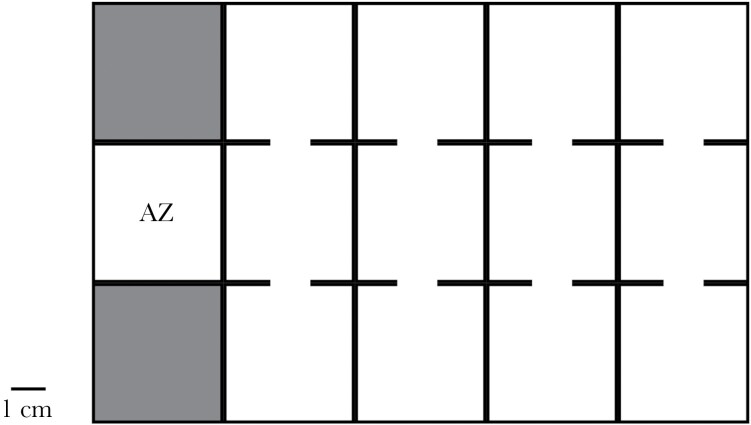
Diagram of the exploration arena. Tadpoles started the trial in the acclimation zone (AZ), spending 10 min behind an opaque barrier. After acclimation, tadpoles were free to explore the central corridor zones and adjacent zones to the left and right over 15 min. Shaded areas represent unused sealed zones.

To quantify individual exploration of a novel context, we filmed focal tadpole movements in a novel arena (dimensions: 29.8 × 19.5 × 4.9 mm; iDesign, UK), filled with 500 ml of aerated tap water and warmed to lab temperature. The arena consisted of an acclimation zone (AZ) which opened to a central corridor with four compartments on both the left- and right-hand sides (Figure 5). To start a trial, we transferred one focal tadpole to the AZ and left them to acclimate for 10 min. We covered the top of the AZ with an opaque barrier to prevent disturbance from the investigator, and during acclimation we sealed the entrance to the corridor with an opaque removeable barrier. After acclimation, the investigator lifted the front portion of the barrier (the top barrier remained in position), providing the tadpole with access to the arena, and the tadpole’s movements were recorded over 15 min. The arena was cleaned between trials using tap water and fresh water was used for each new trial and tadpole.

### Predator risk-taking assay

**Figure 6:**
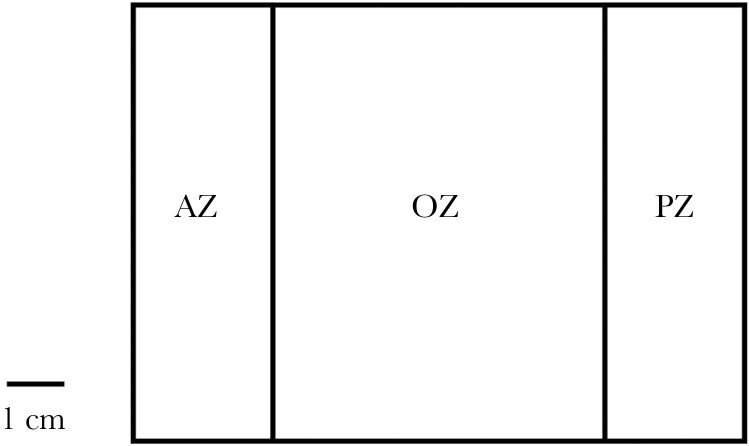
Diagram of the predation arena. AZ represents the AZ where tadpoles acclimated to assay conditions for 10 min. PZ represents the predator zone which contained a live dragonfly larval predator. OZ represents the open zone where the tadpole could explore when the opaque AZ barrier was removed for 15 min. The barrier between the OZ and PZ was transparent and to allow visual predator cues to pass into the OZ.

To quantify predatory risk-taking behavior in a high risk context, we recorded tadpole movements in the presence of visual and olfactory cues from a dragonfly larvae predator (family: Gomphidae) in a novel arena (dimensions: 17 × 12.5 × 4.6; Western Boxes, UK). Each arena consisted of a covered AZ, an open zone (OZ) in which the tadpole could explore and a predator zone (PZ) which was transparent to allow visual cues of the predator in the OZ (Figure 6). To start a trial, a dragonfly larva was placed into the arena PZ and the focal tadpole was placed into the arena AZ. The AZ was sealed with an opaque barrier to allow tadpoles to acclimate. 10 ml of predator conditioned water was also added into the OZ to act as an additional predator olfactory cue after tadpoles and predators were added to the AZ and PZ respectively. Tadpoles were given 10 min to acclimate within the AZ before the barrier between the AZ and OZ was removed, we then recorded tadpole movements over 15 min. The arena was cleaned between trials using tap water and fresh water was used for each new trial and tadpole. Dragon fly larvae were collected from the Aripo Savannah in Trinidad, where both *E. pustulosus* and *L. fuscus* were also observed to co-occur alongside the dragonfly larvae. When not used in assays, we housed the dragonfly larvae in an 11L Perspex tank and we fed them with four *E. pustulosus* tadpoles (which had died of natural causes) each morning.

### Video processing

Post filming, to reduce storage space and increase processing speed, all videos were re-sized to 640 × 360 pixels and the activity and exploration assay videos were reduced from 25 to 1 fps, using the command line tool ffmpeg (Tomar 2006). The predation assay trials were reduced to a higher frame rate of 5 fps to capture the faster movements of tadpoles in this assay. In all three assays, we measured the total distance a tadpole traveled in pixels using a custom-written tracking tool (written by CB) developed in Python v3.0 and using the OpenCV v4.4 library. The tracking tool code can be found on Github (see data availability). In the exploration and predatory risk-taking assays, tadpoles that did not leave the AZ received a distance score of 0.

### Morphological measures

The snout vent length (SVL) of each tadpole was measured in FIJI v2.0 (Schindelin et al. 2012) to the nearest 0.1 mm from the activity assay recordings as a measure of body size. Measurements were taken from each activity trial to give six SVL measurements for each tadpole.

All procedures were approved by the University of Edinburgh ethics committee, under the assessment pwalsh1-0001. Permits to collect *E. pustulosus* and *L. fuscus* were obtained from Trinidad’s Forestry and Wildlife Division.

### Statistical analysis

We estimated the effect of treatment on tadpole body size and tadpole behavior in two separate models using a Bayesian approach.

### Treatment effects on tadpole body size

To estimate the effect of treatment on tadpole body size we fitted a univariate linear mixed model with a Gaussian error distribution (Dingemanse and Dochtermann 2013). We included a fixed effect of treatment (no-competition, conspecific and heterospecific treatment) to estimate the effect the social environment had on the average body size of tadpoles. We also included a fixed effect of trial (fitted as a continuous covariate, coded from 0 to 5) to estimate how body size changed from trial one to six and a random effect of tadpole egg mass ID in the model. To investigate whether treatment affected tadpole growth rates, we included a treatment-specific interaction between trial order (coded from 0 to 5) and size in a random slope model. The random slope model allowed us to determine whether tadpoles in each treatment showed variance amongst individuals in their initial size by fitting a random intercept at trial zero for each tadpole and whether there was variance amongst individuals in their growth rates by fitting a random slope between trial zero and trial five for each tadpole.

### Treatment effects on tadpole behavior

To estimate the effect of social treatment on 1) the population mean behavior, 2) variance among individuals and 3) variance within individuals, we fitted a multivariate generalized linear mixed model (Dingemanse and Dochtermann 2013) of total distance traveled in a familiar context (activity behavior), a novel context (exploration behavior) and predation-risk context (predatory risk-taking behavior). The multivariate model allowed all parameters i–iii to be estimated the total distance traveled in each context simultaneously as well as the pairwise correlations between parameter ii in each context using trial order as the pairing criteria. Given that total distance traveled is a variable constrained to be positive and can be bounded to zero in some contexts, we used a log-normal distribution. As there were a high proportion of exploration and predation-risk-taking assay trials where tadpoles never left the acclimatization zone (54% and 56% of trials respectively), we used a hurdle log-normal distribution for these assays (Hsu and Liu 2008). A hurdle log-normal distribution is in fact a mixture distribution combining a binomial process and a log-normal process. This is adequate for the exploration and predation-risk assays, where the tadpoles decide to leave the AZ or not and then explore the arena. One of the advantages of the hurdle log-normal distribution is that it additionally allowed us to look at a final population level parameter which was iv) the probability that tadpoles remained in the AZ. We used the log-normal distribution for the activity data so that the distance measures in all three contexts could be estimated on the same log scale and aid the comparison of results between the three contexts in the multivariate model.

To estimate population differences in whether tadpoles left the AZ or not for each treatment, we fitted a treatment-specific fixed effect to the hurdle model for the familiar, novel, and predatory risk contexts.

The same fixed effect structure used in the body size univariate model was fitted to the familiar, novel and predator risk contexts in the multivariate model. We fitted two models, one where scaled body size was fitted as a fixed effect and one where scaled body size was not fitted to the model. However, the inclusion of body size did not change the study conclusions and thus was kept in the model. Fitting a fixed effect of treatment allowed us to estimate the overall mean distance tadpoles swam in each treatment and each context.

To estimate the effect of treatment on the variance among individuals in each context, we included a treatment-specific effect for tadpole identity. To estimate the effect of treatment on the variance within individuals we included a treatment-specific fixed effect to the residuals. To estimate the effect of treatment on the probability that tadpoles remained in the AZ, we fitted a treatment-specific fixed effect to the hurdle model for the novel and predator risk contexts.

To determine the effect of treatment on the repeatability of tadpole behavior, a separate repeatability estimate was computed for each treatment in the familiar, novel and predator risk contexts. This gave a total of nine repeatability estimates. Relatabilities were computed using posterior variance estimates of among individual variance and within individual variance obtained from the multivariate mixed model. As such our treatment-specific estimates of repeatability also controlled for body size and trial order, making them estimates of adjusted repeatability (Nakagawa and Schielzeth 2010).

Estimating among individual variance in mean behavior in the familiar, novel and predator risk contexts provided a 3 × 3 covariance matrix allowing us to estimate the individual correlations in mean behavior between each context for each of the three treatments. Given that tadpoles were only exposed to one social treatment, we could not estimate the correlation across treatments at the individual level. We converted all covariance estimates into correlations to aid the interpretation of the results.

To determine whether overall mean body size, variance among individuals in initial body size and variance among individuals in growth rate differed between treatments, we compared the posterior estimates of each parameter across each of the three treatments. To further determine the effect of treatment on behavior, we compared the posterior estimates of mean behavior, variance among individuals, variance within individuals, repeatability, probability of remaining in the AZ and correlations between the average distance swam in each context between the three treatments. Treatment comparisons were calculated as no-competition minus the conspecific treatment, no-competition minus the heterospecific competition treatment and conspecific minus the heterospecific competition. We have reported the posterior mean for each parameter and treatment comparison with the highest posterior density interval (HPDI) at 95%.

All models were fitted using the brms package v2.15 (Bürkner 2018) within (RStudio Team, 2021). Brms displays posterior group effect estimates (variation among individuals, variation within individuals and variation among egg masses) as standard deviations which we converted to variances. Furthermore, as brms displays the posterior residual variance of log-normal and hurdle log-normal distribution models on the log scale, the exponential of the posterior residual variance was taken to obtain estimates of within individual variance on their original scale. This provided us with estimates of among and within individual variance which were on the same scale for estimating repeatability. Finally, we converted posterior hurdle model estimates from the logit scale to probability estimates to aid interpretation.

The univariate and multivariate models used four chains with 8500 iterations and a burn in period of 1000 iterations and a thinning interval of 100. We used uninformative or weak priors on all parameters (Gelman et al. 2013) which included wide normal priors for fixed effects, Half-Student priors for variance parameters and LKJ correlation priors for correlations. The models met all assumptions on convergence and autocorrelation and posterior predictive checks were used to determine if the model fitted the observed data (Gelman et al. 2013).

## RESULTS

### The effect of treatment on body size

**Figure 7:**
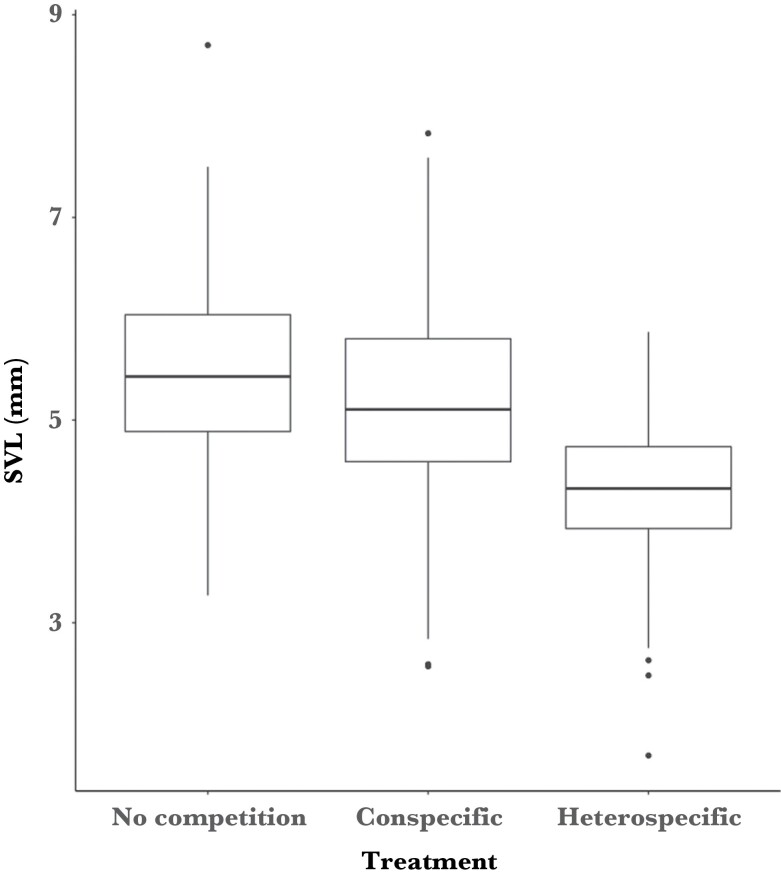
Box and whisker plot of mean tadpole snout vent length (SVL) in the no-competition, conspecific, and heterospecific treatment groups.

Tadpoles from the no-competition treatment were larger than tadpoles from the heterospecific treatment and marginally larger than tadpoles in the conspecific treatment (Tables 1 and 2, Figure 7 and Supplementary Figure S1). Tadpoles experiencing conspecific competition were also larger than individuals experiencing heterospecific competition (Tables 1 and 2, Figure 7 and Supplementary Figure S1). Tadpole body size increased from trial 1 though to trial 6 and there was no among individual variance associated with Egg Mass ID (Table 1). Across all three treatments, tadpoles showed among individual variance in their initial body size (Table 1, Supplementary Figure S2) but this variance did not differ between treatments (Table 2, Supplementary Figure S2). Tadpoles did not show among individual variance in their growth rates in any treatment (Table 1, Supplementary Figure S3) and this variance did not change between treatments (Table 2, Supplementary Figure S3).

**Table 1:** Posterior estimates for treatment effects on mean body size (population means), variance among individuals in their initial body size and variance among individuals in their growth rate over the course of the six trials

	Body size		
	Mean	95% CI	
		2.5	95.7
Population means			
No-competition	5.285	4.988	5.588
Conspecific	5.014	4.714	5.339
Heterospecific	4.168	3.865	4.478
Trial	0.056	0.035	0.079
Variance among egg masses			
Egg mass ID	0.149	0.000	0.441
Variance among individuals in initial body size			
No-competition	0.288	0.105	0.504
Conspecific	0.404	0.204	0.621
Heterospecific	0.406	0.162	0.705
Variance among individuals in change in body size			
No-competition	0.004	0.000	0.010
Conspecific	0.008	0.000	0.017
Heterospecific	0.006	0.000	0.014

Also displayed are population mean estimates for trial and variance in body size among egg masses. Estimates are displayed alongside their 95% credible intervals (CI).

**Table 2:** Posterior estimates of the treatment differences on mean body size, variance in initial body size among individuals and variance in the change in body size over the six trials

	Body size		
	Mean	95% CI	
		2.5	95.7
Population means			
No-competition—conspecific	0.273	0.040	0.494
No-competition—heterospecific	1.117	0.869	1.377
Conspecific—heterospecific	0.846	0.584	1.105
Variance among individuals in initial body size			
No-competition—conspecific	0.148	0.000	0.349
No-competition—heterospecific	0.194	0.000	0.495
Conspecific—heterospecific	0.156	0.000	0.395
Variance among individuals in change in body size			
No-competition—conspecific	0.005	0.000	0.013
No-competition—heterospecific	0.004	0.000	0.011
Conspecific—heterospecific	0.005	0.000	0.013

Estimates are displayed alongside their 95% credible intervals (CI).

### The effect of treatment on behavior

At a population level, treatment did not affect the average distance tadpoles swam in a familiar context (Tables 3 and 4, Supplementary Figure S4). In a novel context, tadpoles in the no-competition treatment were more exploratory than tadpoles in both the heterospecific and conspecific treatments (Tables 3 and 4, Supplementary Figure S4). In a predator risk context, tadpoles in the no-competition treatment swam further than tadpoles in the conspecific treatment (Tables 3 and 4, Supplementary Figure S4).

**Table 3:** Posterior estimates for the treatment effects on population means, variance among individuals, variance within individuals and the probability that tadpoles remained in the acclimation zone for activity, exploration and predatory risk-taking (predation) behaviors

	Activity			Exploration			Predation		
	Mean	95% CI		Mean	95% CI		Mean	95% CI	
		2.5	97.5		2.5	97.5		2.5	97.5
Population means									
No-competition	6.228	5.849	6.624	6.819	6.391	7.230	6.141	5.740	6.521
Conspecific	6.221	5.833	6.550	6.212	5.744	6.725	5.849	5.422	6.273
Heterospecific	6.319	5.907	6.744	5.854	5.367	6.355	5.852	5.443	6.289
SVL	–0.088	–0.225	0.052	–0.227	–5.000	–0.069	–0.010	–0.200	0.187
Trial	–0.114	–0.173	–0.061	–0.186	–0.267	–0.089	–0.068	–0.154	0.025
Variance among egg masses									
Egg mass ID	0.157	0.000	0.429	0.137	0.000	0.443	0.054	0.000	0.194
Variance among individuals									
No-competition	0.193	0.000	0.455	0.129	0.000	0.440	0.144	0.000	0.483
Conspecific	0.175	0.000	0.389	0.863	0.000	1.745	0.493	0.000	1.181
Heterospecific	0.686	0.303	1.176	0.403	0.000	1.008	0.127	0.000	0.384
Variance within individuals									
No-competition	2.796	2.332	3.285	2.773	2.247	3.418	2.703	2.142	3.360
Conspecific	1.770	1.465	2.073	3.466	2.574	4.455	2.788	2.026	3.520
Heterospecific	2.316	1.897	2.724	2.736	2.045	3.446	2.773	2.195	3.387
Probability remained in acclimation zone									
No-competition	NA	NA	NA	0.375	0.321	0.426	0.450	0.394	0.505
Conspecific	NA	NA	NA	0.512	0.458	0.565	0.508	0.457	0.560
Heterospecific	NA	NA	NA	0.432	0.380	0.489	0.416	0.362	0.472

Also displayed are population mean estimates for body size (SVL) and trial number as well as variance among egg masses. Estimates are displayed alongside their 95% credible intervals (CI).

**Table 4:** Posterior estimates of the treatment differences on population means, variance among individuals, variance within individuals and the probability that tadpoles remained in the acclimation zone for activity, exploration and predatory risk-taking (predation) behaviors

	Activity			Exploration			Predation		
	Mean	95% CI		Mean	95% CI		Mean	95% CI	
		2.5	97.5		2.5	97.5		2.5	97.5
Population means									
No-competition—conspecific	0.121	0.000	0.297	0.607	0.159	1.111	0.312	0.001	0.662
No-competition—heterospecific	0.178	0.000	0.438	0.965	0.457	1.450	0.315	0.000	0.685
Conspecific—heterospecific	0.173	0.000	0.418	0.383	0.000	0.804	0.186	0.000	0.469
Variance among individuals									
No-competition—conspecific	0.145	0.000	0.368	0.751	0.000	1.640	0.411	0.000	1.062
No-competition—heterospecific	0.499	0.015	0.963	0.333	0.000	0.918	0.149	0.000	0.439
Conspecific—heterospecific	0.513	0.000	0.976	0.594	0.000	1.465	0.409	0.000	1.054
Variance within individuals									
No-competition—conspecific	1.026	0.462	1.602	0.749	0.001	1.700	0.408	0.001	0.990
No-competition—heterospecific	0.500	0.000	1.013	0.379	0.000	0,938	0.352	0.000	0.876
Conspecific—heterospecific	0.549	0.000	0.999	0.794	0.001	1.786	0.405	0.001	0.985
Probability remained in acclimation zone									
No-competition—conspecific	NA	NA	NA	0.136	0.058	0.206	0.060	0.000	0.122
No-competition—heterospecific	NA	NA	NA	0.059	0.000	0.123	0.043	0.000	0.101
Conspecific—heterospecific	NA	NA	NA	0.080	0.003	0.146	0.092	0.014	0.164

Estimates are displayed alongside their 95% credible intervals (CI).

In a familiar context, only tadpoles in the heterospecific treatment show variance among individuals in the distance swam and this variance was found to be larger than that observed in the no-competition treatment (Tables 3 and 4, Supplementary Figure S5). Tadpoles did not show among individual variation in the distance swam in the novel and predator risk contexts (Table 3, Supplementary Figure S5) and these did not change between treatments (Table 4, Supplementary Figure S5).

Concerning patterns of within individual (residual) variance, tadpoles showed within individual variance in the distance swam across all three contexts (Table 3, Supplementary Figure S6). In a familiar context, tadpoles in the conspecific treatment were more consistent in the distance they swam than tadpoles in the no-competition treatment (Table 4, Supplementary Figure S6). In novel and predator risk contexts, tadpoles in the conspecific treatment were less consistent in the distance they swam than tadpoles in the no-competition and heterospecific treatments (Table 4, Supplementary Figure S6).

In a familiar context, the distance tadpoles swam was only repeatable within the heterospecific treatment and was greater than the amount of repeatability observed within the no-competition treatment (Tables 5 and 6, Supplementary Figure S7). The distance tadpoles swam was not repeatable in any treatment within novel and predator risk contexts and did not change between treatments (Tables 5 and 6, Supplementary Figure S7).

**Table 5:** Posterior estimates of repeatability for activity, exploration and predatory risk-taking behaviors (predation) in the no-competition, conspecific, and heterospecific treatments

	Activity			Exploration			Predation		
	Mean	95% CI		Mean	95% CI		Mean	95% CI	
		2.5	97.5		2.5	97.5		2.5	97.5
No-competition	0.064	0.000	0.148	0.044	0.000	0.140	0.050	0.000	0.166
Conspecific	0.089	0.000	0.187	0.195	0.000	0.364	0.146	0.000	0.319
Heterospecific	0.226	0.107	0.345	0.125	0.000	0.293	0.043	0.000	0.127

Estimates are displayed alongside their 95% credible intervals (CI).

**Table 6:** Posterior estimates of the treatment differences on repeatability for activity, exploration, and predatory risk-taking (predation) behaviors

	Activity			Exploration			Predation		
	Mean	95% CI		Mean	95% CI		Mean	95% CI	
		2.5	97.5		2.5	97.5		2.5	97.5
No-competition—conspecific	0.060	0.000	0.150	0.159	0.000	0.331	0.119	0.000	0.290
No-competition—heterospecific	0.163	0.016	0.296	0.101	0.000	0.266	0.050	0.000	0.146
Conspecific—heterospecific	0.140	0.000	0.280	0.124	0.000	0.294	0.118	0.000	0.285

Estimates are displayed alongside their 95% credible intervals (CI).

In novel contexts, tadpoles in the conspecific treatment were the most likely to remain in the AZ compared to tadpoles in the no-competition and heterospecific treatments (Tables 3 and 4, Supplementary Figure S8). The predator risk context was similar, with tadpoles in the conspecific treatment being more likely to remain in the AZ than in the heterospecific treatment (Tables 3 and 4, Supplementary Figure S8).

There was no correlation in the average distance individual tadpoles swam between contexts (Supplementary Table S1). These correlations also did not differ between treatment regimes (Supplementary Table S2).

## DISCUSSION

We exposed tadpoles to no-competition, conspecific competition and heterospecific competition during their development and measured the total distance they swam over multiple trials in familiar, novel and predator risk contexts. We found that conspecific and heterospecific competition resulted in different patterns of among and within individual variation which impacted the repeatability of behavior across the three contexts. Within a familiar context and under heterospecific competition, we found that there was among individual variance in the average distance tadpoles swam, leading to repeatable differences in activity behavior. However, under conspecific competition, there was a decrease in the variance within individuals, but this did not lead to any increase in behavioral repeatability. There was also an increase in within individual variance in the distance swam in novel and predator risk contexts. Our results show that the impact of competition on among and within individual variance as well as behavioral repeatability is dependent on competitor species identity and is context specific.

Ecological theory predicts that individuals can alleviate competition for resources through feeding specialization (MacArthur 1958; Bolnick et al. 2003). Changes in foraging behavior may also be important in promoting co-existence where there is high resource overlap (Pfennig et al. 2006, 2007; Kent and Sherry 2020; Sherry et al. 2020). In our experiment, conspecific, and heterospecific competition affected the among and within individual components of tadpole activity behavior independently and this suggests that behavioral mechanisms for reducing conflict over contested resources may be different for single and multi-species interactions. The increase in among individual variance and repeatability under heterospecific competition may have provided a mechanism by which food resources could be consistently partitioned between focal and non-focal tadpoles (Sherry et al. 2020; Prati et al. 2021). Therefore, the change of behavior in the presence of a heterospecific competitor may promote diversification in how resources are acquired and may be a mechanism which promotes species co-existence (Pfennig et al. 2006, 2007). For example, by diverging in the average distance they swam, focal tadpoles may have adjusted their foraging behavior to target the single dietary resource, fish flakes, via different behavioral mechanisms and reduce conflict with *L. fuscus* individuals (e.g. foraging on the water surface vs at the bottom of the tank). Equally, among individual variance in activity behavior may also reflect differences in the susceptibility of *E. puslulosus* tadpoles to heterospecific competition due to individual differences in body size or metabolic rate (Careau et al. 2008; Biro and Stamps 2010; Kelleher et al. 2017). For example, less competitive individuals may have had to forage more intensely than more competitive individuals. Alternatively, differences in morphology and/or behavior among *L. fucus* individuals may have contributed to the diversity of behavioral responses observed in the focal *E. pustulosus* tadpoles via indirect effects (Wolf et al. 1998; Wilson et al. 2009; Jäger et al. 2019).

In the conspecific treatment, the decrease in within individual variance without the corresponding change in behavioral repeatability suggests that individuals were not partitioning resources through behavioral specialization. A more likely explanation is that the increased consistency in swimming movements was to allow focal tadpoles to behave more similarly to the non-focal tadpole (Herbert-Read et al. 2013). This may be beneficial for promoting increases in foraging gains through group foraging (Rook and Penning 1991; Rands et al. 2014), reduce the costs of locomotion (Marras et al. 2015) or provide increased protection from predators (Landeau and Terborgh 1986; Szulkin et al. 2006). In fish shoals, individuals may conform in their behavior to produce coordinated changes in direction and bursts of speed (Jolles et al. 2018, 2020; Sankey et al. 2019). This may be mediated by a decrease in behavioral variation both between each other and within themselves (Webster et al. 2007; Magnhagen and Bunnefeld 2009; Herbert-Read et al. 2013). Whilst shoaling behavior has not been reported in *E. pustulosus* tadpoles, other larvae of anuran species such as cane toads (*Rhinella marina*) and common toads (*Bufo bufo*) are known to form dense aggregations (Wassersug et al. 1981; Griffiths and Foster 1998) where behavioral conformity may be important. To elucidate whether the decrease in within individual variance in response to conspecifics was driven by competition over resources or behavioral conformity, future studies could record the behavior of both focal and non-focal individuals. If both individuals show similar patterns of behavior and low within individual variance, this will indicate behavioral conformity over behavioral specialization.

In addition to the species identity of a competitor impacting the variance in behavior among and within individuals, we found the effect of competitive treatment was highly context dependent. In particular, the level of within and among individual variation in the distance swam in home tanks had no relation to the level of among or within individual variation within novel or high predation-risk contexts. Consequently, studies which only consider individual level behavioral responses in a single context are likely to miss elements of behavioral variation that could be relevant in other ecological contexts. There was also no evidence that competition could alter the structure of behavioral syndromes within a population.

Since low numbers of tadpoles left the AZ in the novel and predator risk contexts, our power to detect differences in the repeatability of behavior between treatments was limited (Martin et al. 2011; Dingemanse and Dochtermann 2013). Nevertheless, tadpoles in the conspecific treatment were the least likely to leave the AZ across both the novel and predator risk contexts compared to tadpoles housed in isolation or with a heterospecific. This provides further support for our findings that behavioral responses to competition were both context specific and dependent on the species of the competitor. Jolles et al (2016) suggested that testing fish in isolation when they had previously been housed in groups may induce stress (Gallup and Suarez 1980) compared to individuals which had always been housed alone. This may contribute to a reduced tendency for individuals to take risks (Jolles et al. 2016). A similar mechanism could explain the high number of tadpoles in the conspecific treatment which remained in the AZ in the present study, compared to the increased exploration levels in the no-competition treatment.

When tadpoles did leave the AZ in the conspecific treatment, individuals were found to be less consistent in novel and predator risk contexts compared to the no-competition and heterospecific treatments. Low consistency in behavior has been suggested as an adaptive strategy to reduce an individual’s susceptibility to predation (Maye et al. 2007; Stamps et al. 2012; Biro and Adriaenssens 2013; Briffa 2013). Consequently, when tadpoles took risks to leave the AZ, their increased unpredictability in swimming movements may have been to offset the increased chance of predation in riskier contexts.

## CONCLUSIONS

This study shows that both conspecific and heterospecific competition can impact individual differences in behavior but may be mediated through different mechanisms, affecting among and within sources of individual variation independently. As highlighted by the effect of conspecific competition on the consistency of behavior in familiar and novel contexts, this study also demonstrates that responses to competition is context dependent. Future investigations should consider how individual variation in behavior may change in response to early life conditions depending on the behaviors and contexts investigated.

## Supplementary Material

arac109_suppl_Supplementary_Material

## Notes

We would like to thank Greig Muir, Professor Paul Hoskisson, Renoir Auguste and members of the 2019 Glasgow Trinidad Expedition for their help in collecting nests and/or running behavioral assays in this study.
