# Supplementary material for: The effect of heterospecific and conspecific competition on inter-individual differences in tungara frog tadpole (Engystomops pustulosus) behavior
Source: Behav Ecol. 2023 Jan 7;34(2):210–22. doi: 10.1093/beheco/arac109 (PMC10047633; doi:10.1093/beheco/arac109)
Supplement: arac109_suppl_Supplementary_Material [file arac109_suppl_supplementary_material.pdf]

Supplementary materials

Table of Contents

1. **Comparison of tadpole mortality rates across treatments 1**
2. **Effect of treatment on tadpole body size 2**
3. **Effect of treatment on tadpole behaviour 5**
4. **Effect of treatment on correlations between assays at the among individual level. 10**
5. **Treatment differences between assay correlations at the among individual level. 10**

1. Comparison of tadpole mortality rates across treatments

In total, across the four experimental batches, we collected data from 54 tadpoles in the no competition treatment, 56 focal tadpoles in the conspecific treatment and 51 tadpoles in the heterospecific treatment. 40 tadpoles died during the experiment which are not included in the final tadpole count. This included 10 tadpoles in each of the no competition and conspecific treatments and 20 tadpoles in the heterospecific treatment. This highlighted the increased severity of the heterospecific treatment in comparison to the no-competition (three sample proportion test with Bonferroni-Holm adjustment: X-squared = 8.26, df = 2, p = 0.038) and conspecific (p = 0.038) treatment groups. There was no difference between the number of tadpoles which died in the no-competition and conspecific treatment groups (p = 0.50).

2. Effect of treatment on tadpole body size

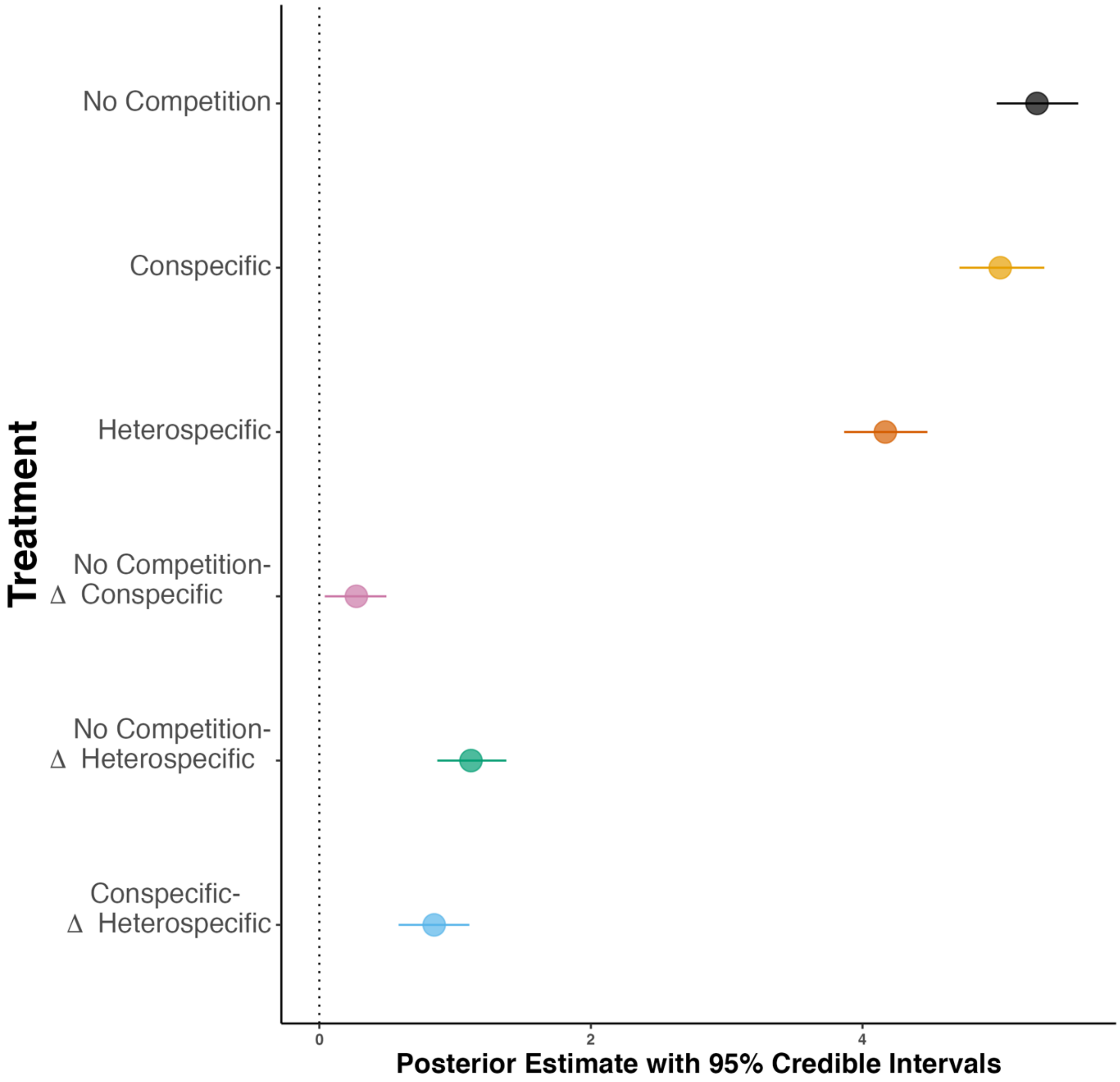

Figure S1. Treatment effects on tadpole mean body size. Points indicate posterior estimates for mean values and associated 95% credible intervals. Estimates are displayed for No Competition (black), Conspecific (orange) and Heterospecific (red) treatment groups. The contrasts between treatments are displayed as the difference between No competition and Conspecific (purple), No Competition and Heterospecific (green) and Conspecific and Heterospecific (blue) treatment groups. Contrasts are displayed as absolute values.

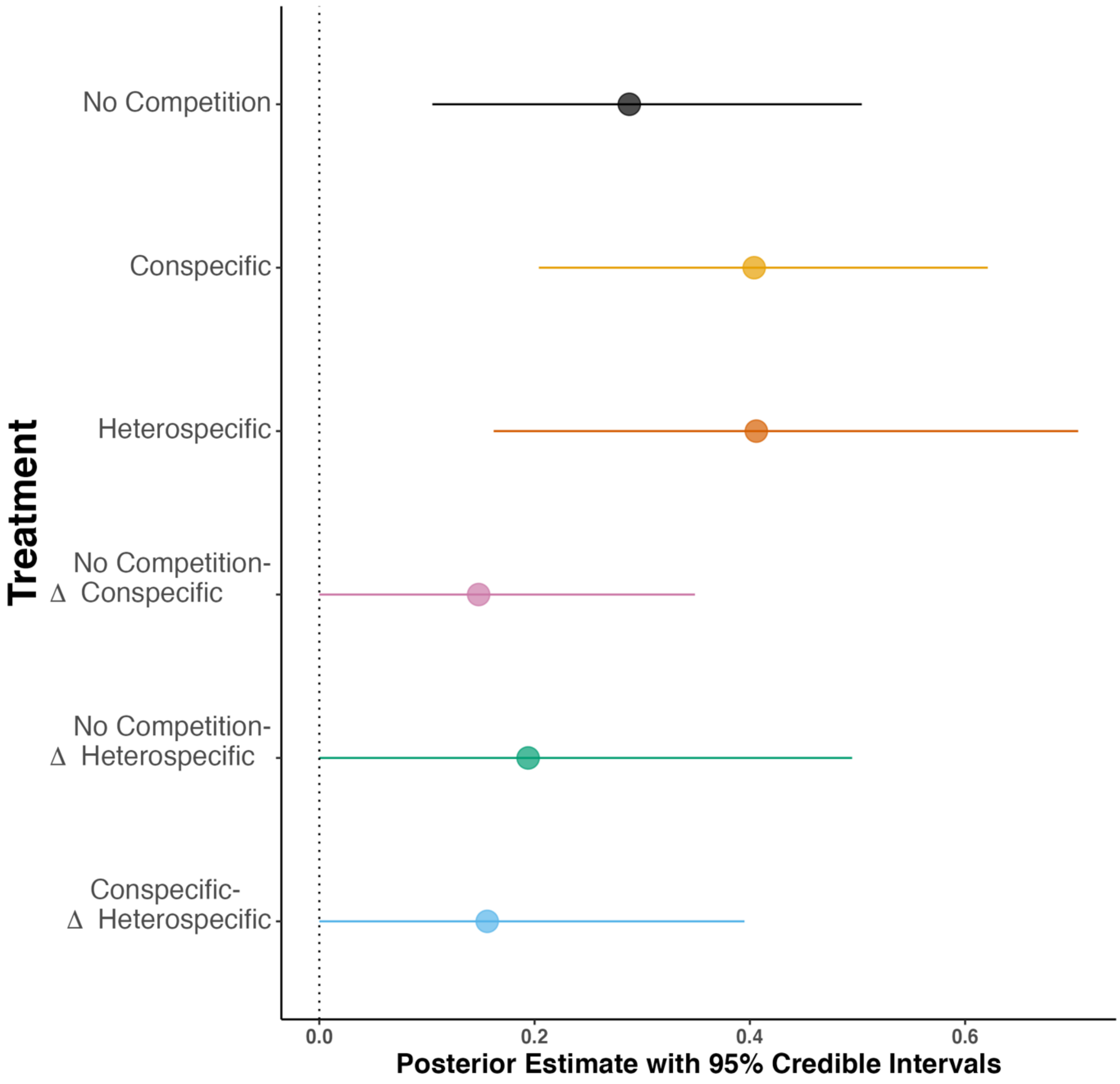

Figure S2. Treatment effects on variance among individuals in their initial body size. Points indicate posterior estimates for mean values and associated 95% credible intervals. Estimates are displayed for No Competition (black), Conspecific (orange) and Heterospecific (red) treatment groups. The contrasts between treatments are displayed as the difference between No competition and Conspecific (purple), No Competition and Heterospecific (green) and Conspecific and Heterospecific (blue) treatment groups. Contrasts are displayed as absolute values.

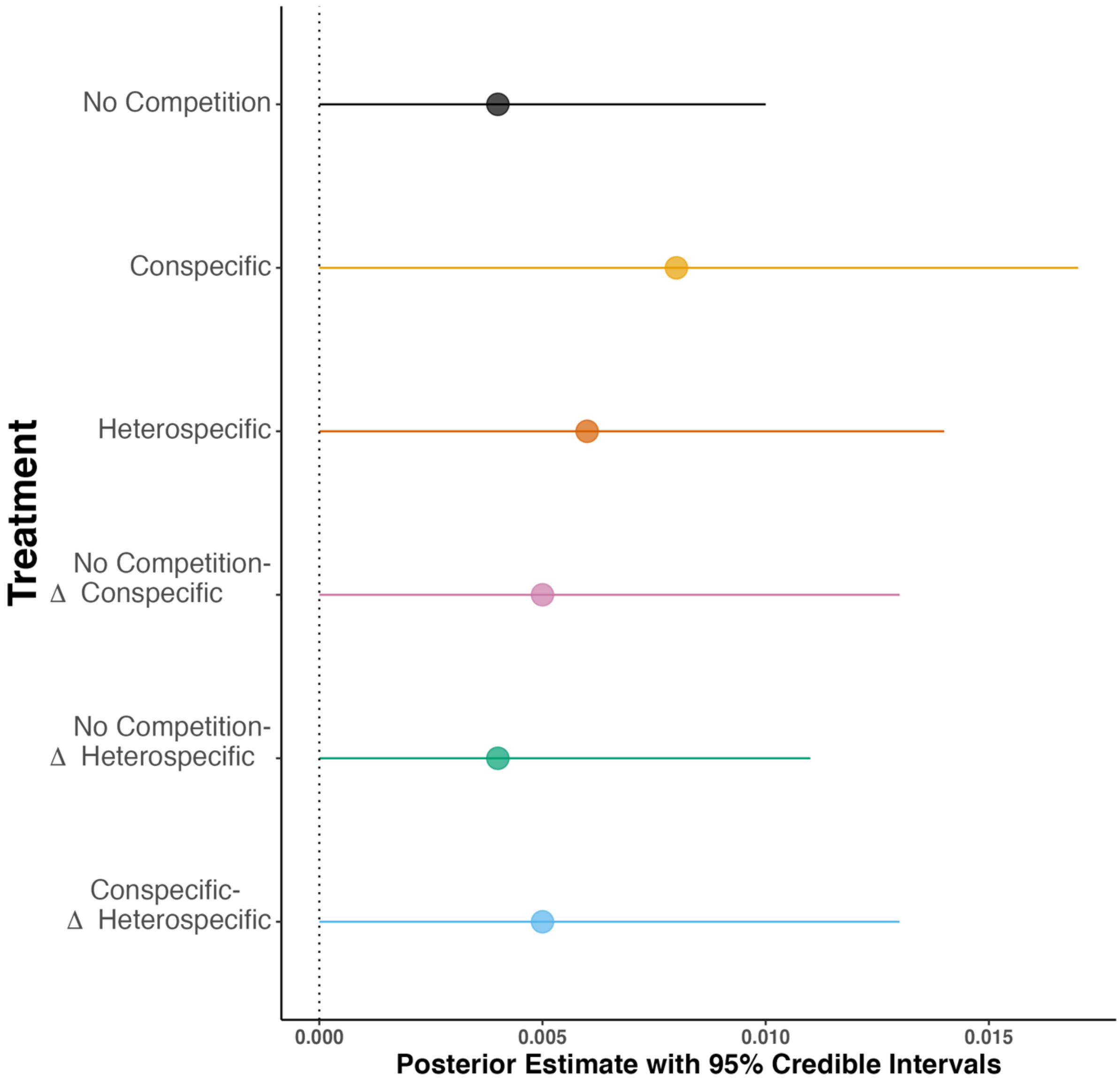

Figure S3. Treatment effects on variance among individual in their growth rates. Points indicate posterior estimates for mean values and associated 95% credible intervals. Estimates are displayed for No Competition (black), Conspecific (orange) and Heterospecific (red) treatment groups. The contrasts between treatments are displayed as the difference between No competition and Conspecific (purple), No Competition and Heterospecific (green) and Conspecific and Heterospecific (blue) treatment groups. Contrasts are displayed as absolute values.

3. Effect of treatment on tadpole behaviour

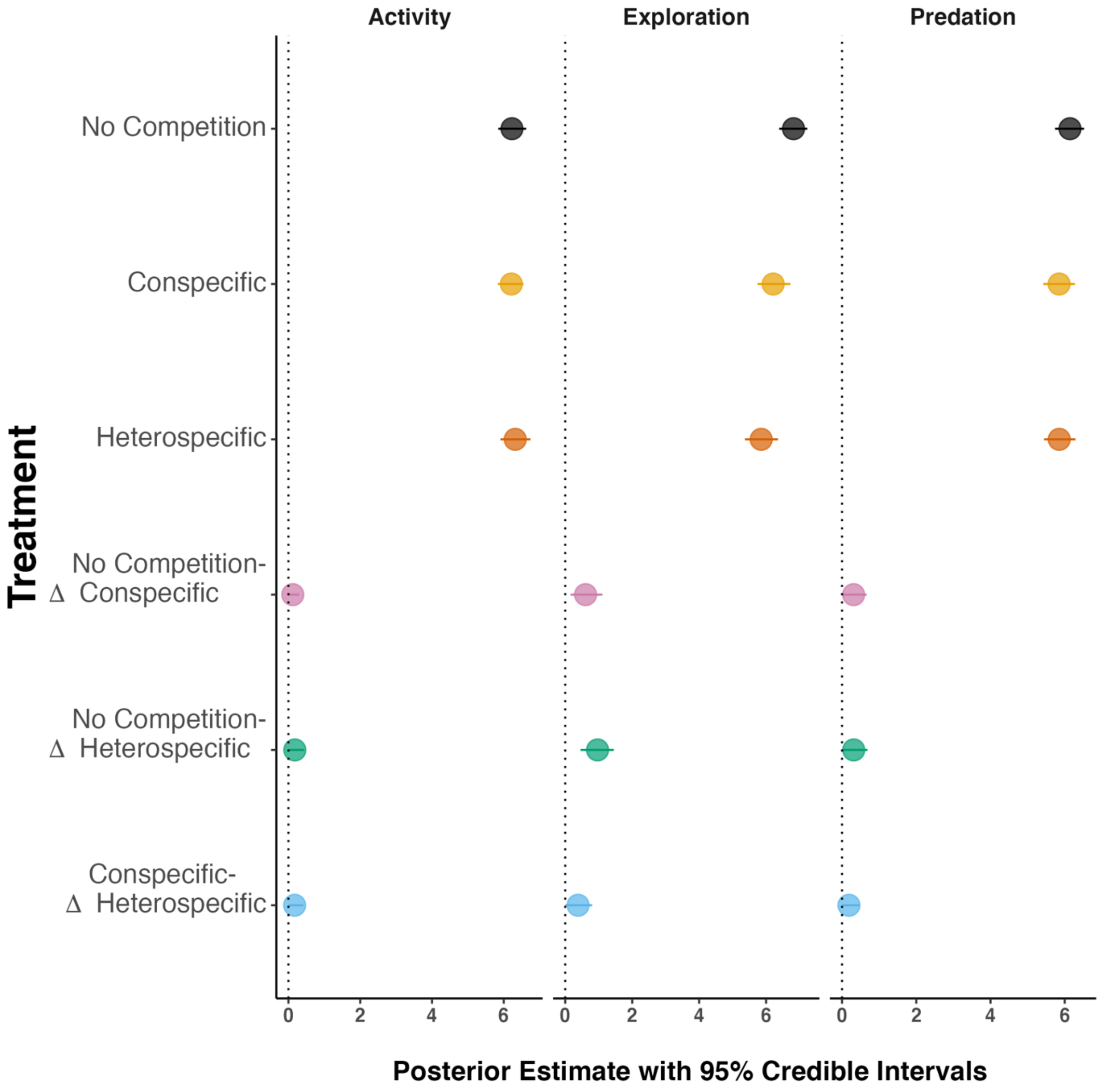

Figure S4. Treatment effects on the overall mean activity, exploration and predatory risk-taking behaviors. Points indicate posterior estimates for mean values and associated 95% credible intervals. Estimates are displayed for No Competition (black), Conspecific (orange) and Heterospecific (red) treatment groups. The contrasts between treatments are displayed as the difference between No competition and Conspecific (purple), No Competition and Heterospecific (green) and Conspecific and Heterospecific (blue) treatment groups. Contrasts are displayed as absolute values.

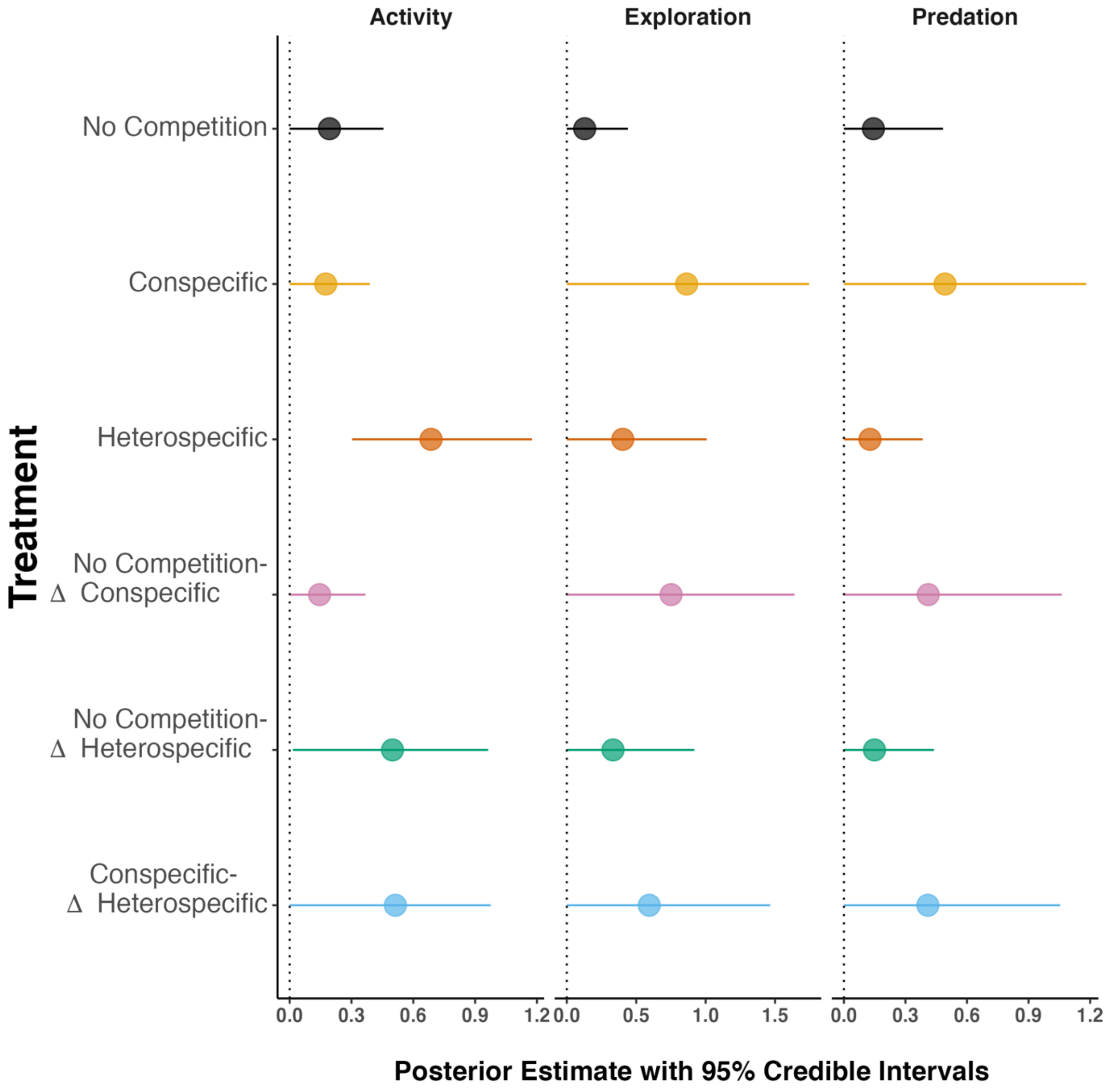

Figure S5. Treatment effects on variance among individuals, for activity, exploration and predatory risk-taking behaviors. Points indicate posterior estimates for mean values and associated 95% credible intervals. Estimates are displayed for No Competition (black), Conspecific (orange) and Heterospecific (red) treatment groups. The contrasts between treatments are displayed as the difference between No competition and Conspecific (purple), No Competition and Heterospecific (green) and Conspecific and Heterospecific (blue) treatment groups. Contrasts are displayed as absolute values.

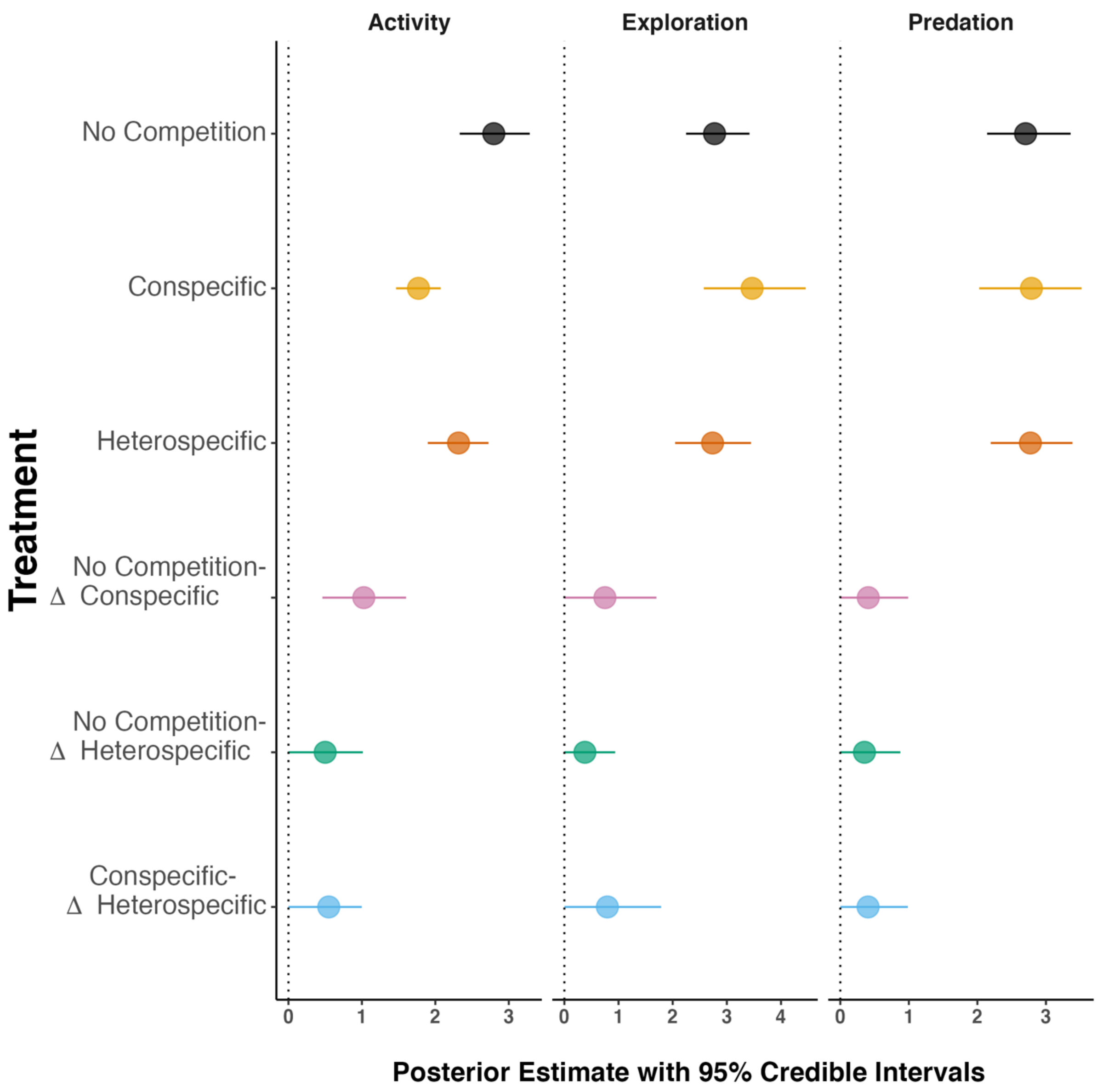

Figure S6. Treatment effects on variance within individuals, for activity, exploration and predatory risk-taking behaviors. Points indicate posterior estimates for mean values and associated 95% credible intervals. Estimates are displayed for No Competition (black), Conspecific (orange) and Heterospecific (red) treatment groups. The contrasts between treatments are displayed as the difference between No competition and Conspecific (purple), No Competition and Heterospecific (green) and Conspecific and Heterospecific (blue) treatment groups. Contrasts are displayed as absolute values.

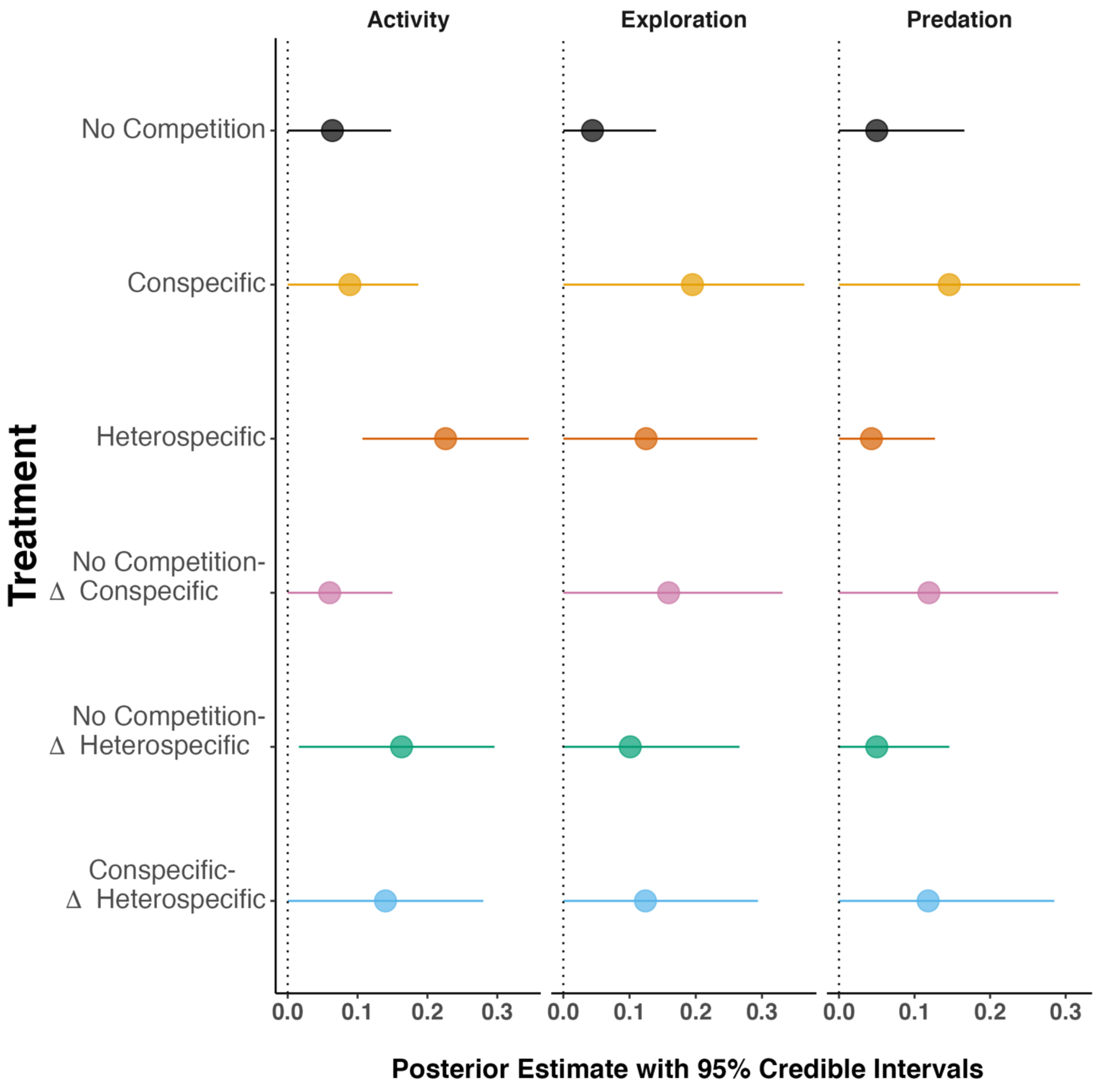

Figure S7. Treatment effects on the repeatability of activity, exploration and predatory risk-taking behaviors. Points indicate posterior estimates for mean values and associated 95% credible intervals. Estimates are displayed for No Competition (black), Conspecific (orange) and Heterospecific (red) treatment groups. The contrasts between treatments are displayed as the difference between No competition and Conspecific (purple), No Competition and Heterospecific (green) and Conspecific and Heterospecific (blue) treatment groups. Contrasts are displayed as absolute values.

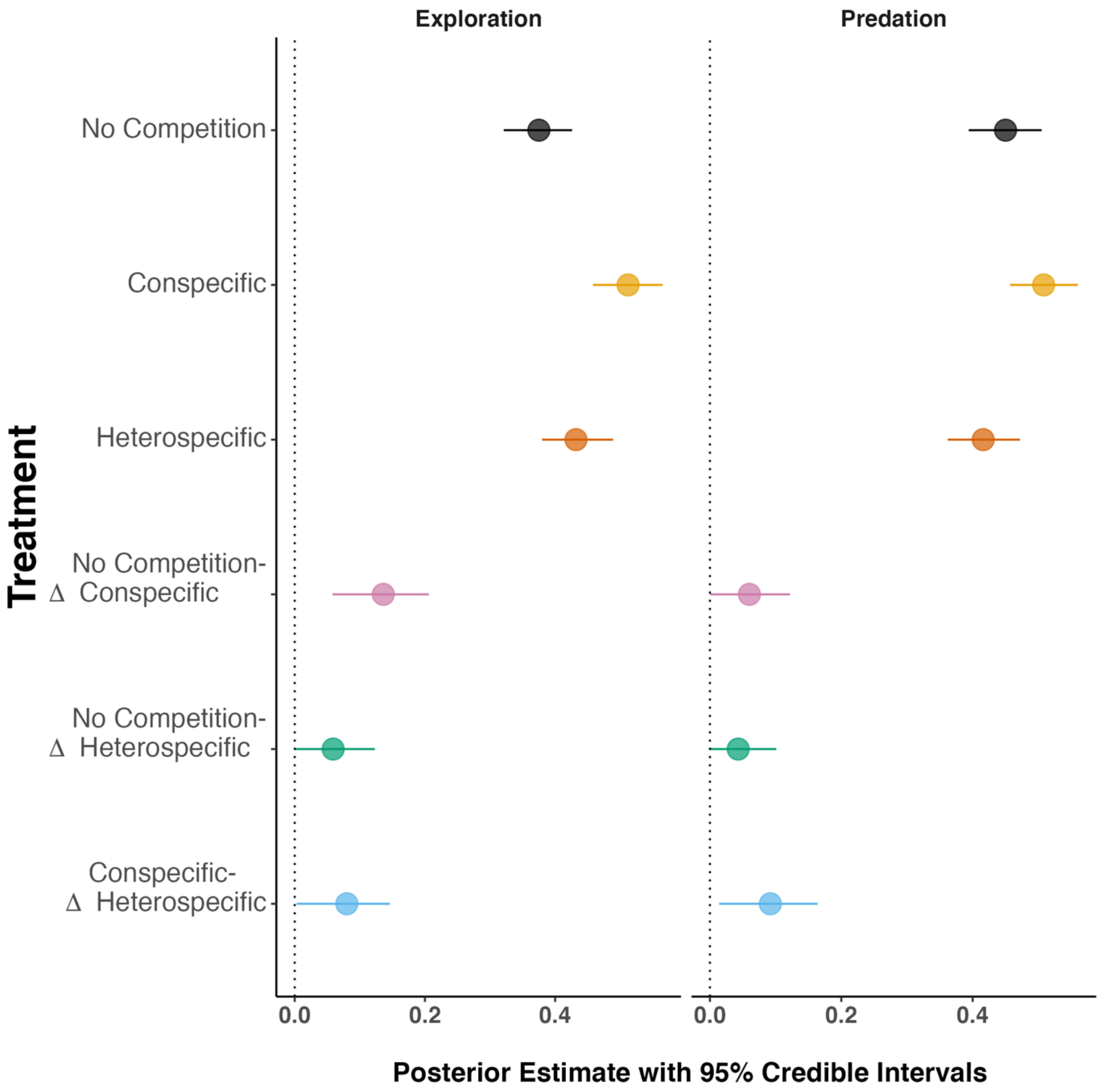

Figure S8. Treatment effects on the probability of remaining in the acclimation zone for the exploration and predatory risk-taking behaviors. Points indicate posterior estimates for mean values and associated 95% credible intervals. Estimates are displayed for No Competition (black), Conspecific (orange) and Heterospecific (red) treatment groups. The contrasts between treatments are displayed as the difference between No competition and Conspecific (purple), No Competition and Heterospecific (green) and Conspecific and Heterospecific (blue) treatment groups. Contrasts are displayed as absolute values.

4. Effect of treatment on correlations between assays at the among individual level.

Table S1. Posterior correlation parameter estimates for among individual correlations in mean behaviour and associated 95% credible intervals (CI). Names of parameters starting with "Act", "Exp" and "Pred" refer to activity, exploration and predation assays respectively. The term VI refers to among individual variance in mean behaviour.

	No Competition			Conspecific			Heterospecific		
		95% CI			95% CI			95% CI	
	Mean	2.5%	97.5%	Mean	2.5%	97.5%	Mean	2.5%	97.5%
ExpVI_ActVI	-0.178	-0.969	0.666	-0.112	-0.799	0.597	0.162	-0.473	0.780
PredVI_ActVI	-0.168	-0.950	0.700	-0.162	-0.874	0.636	0.381	-0.406	0.988
PredVI_ExpVI	-0.011	-0.834	0.873	0.252	-0.425	0.920	0.039	-0.802	0.888

5. Treatment differences between assay correlations at the among individual level.

Table S2. Posterior correlation parameter estimates for the difference in among individual correlations in mean behaviour and associated 95% credible intervals (CI) for each treatment comparison. Names of parameters starting with "Act", "Exp" and "Pred" refer to activity, exploration and predation assays respectively. The term VI refers to among individual variance in mean behaviour.

	No Competition-Conspecific			No Competition-Heterospecific			Conspecific-Heterospecific		
		95% CI			95% CI			95% CI	
	Mean	2.5%	97.5%	Mean	2.5%	97.5%	Mean	2.5%	97.5%
ExpVI_ActVI	-0.066	-1.176	1.046	-0.340	-1.351	0.756	-0.274	-1.216	0.679
PredVI_ActVI	-0.006	-1.230	1.084	-0.549	-1.615	0.672	-0.543	-1.615	0.559
PredVI_ExpVI	-0.263	-1.397	0.871	-0.050	-1.249	1.285	0.213	-0.912	1.290
